# Intervention in Biological Phenomena via Feedback Linearization

**DOI:** 10.1155/2012/534810

**Published:** 2012-11-06

**Authors:** Mohamed Amine Fnaiech, Hazem Nounou, Mohamed Nounou, Aniruddha Datta

**Affiliations:** ^1^Electrical and Computer Engineering Program, Texas A&M University at Qatar, P.O. Box 23874, Doha, Qatar; ^2^Chemical Engineering Program, Texas A&M University at Qatar, P.O. Box 23874, Doha, Qatar; ^3^Department of Electrical and Computer Engineering, Texas A&M University, College Station, TX 77843, USA

## Abstract

The problems of modeling and intervention of biological phenomena have captured the interest of many researchers in the past few decades. The aim of the therapeutic intervention strategies is to move an undesirable state of a diseased network towards a more desirable one. Such an objective can be achieved by the application of drugs to act on some genes/metabolites that experience the undesirable behavior. For the purpose of design and analysis of intervention strategies, mathematical models that can capture the complex dynamics of the biological systems are needed. S-systems, which offer a good compromise between accuracy and mathematical flexibility, are a promising framework for modeling the dynamical behavior of biological phenomena. Due to the complex nonlinear dynamics of the biological phenomena represented by S-systems, nonlinear intervention schemes are needed to cope with the complexity of the nonlinear S-system models. Here, we present an intervention technique based on feedback linearization for biological phenomena modeled by S-systems. This technique is based on perfect knowledge of the S-system model. The proposed intervention technique is applied to the glycolytic-glycogenolytic pathway, and simulation results presented demonstrate the effectiveness of the proposed technique.

## 1. Introduction

Biological systems are complex processes with nonlinear dynamics. S-systems are proposed in [1, 2] as a canonical nonlinear model to capture the dynamical behavior of a large class of biological phenomena [3, 4]. They are characterized by a good tradeoff between accuracy and mathematical flexibility [5]. In this modeling approach, nonlinear systems are approximated by products of power-law functions which are derived from multivariate linearization in logarithmic coordinates. It has been shown that this type of representation is a valid description of biological processes in a variety of settings. S-systems have been proposed in the literature to mathematically capture the behavior of genetic regulatory networks [6–13]. Moreover, the problem of estimating the S-system model parameters, the rate coefficients and the kinetic orders, has been addressed by several researchers [12, 14–16]. In [17], the authors studied the controllability of S-systems based on feedback linearization approach.

Recently, the authors in [18] developed two different intervention strategies, namely, indirect and direct, for biological phenomena modeled by S-systems. The goal of these intervention strategies is to transfer the target variables from an initial steady-state level to a desired final one by manipulating the control variables. The complexity of the nonlinear biological models led researchers to focus on nonlinear control approaches, such as sliding mode control that was introduced in [19].

A basic problem in control theory is how to use feedback in order to modify the original internal dynamics of nonlinear systems to achieve some prescribed behavior [20]. In particular, feedback linearization can be used for the purpose of imposing, on the associated closed-loop system, a desired behavior of some prescribed autonomous linear system. When the system to be controlled is linear time-invariant system, this is known as the problem of pole placement, while in the more general case of nonlinear systems, this is known as the problem of feedback linearization [21, 22]. Significant advances have been made in the theory of nonlinear state feedback control, such as feedback linearization and input-output decoupling techniques [21, 22]. The state feedback linearization technique has been widely utilized in many applications. For example, the authors in [23] have used feedback linearization in cancer therapy, where full knowledge of the state and parameter vectors is assumed to transform a multiinput multioutput nonlinear system into a linear and controllable one using nonlinear state feedback. Then, linear control techniques can be applied for the resulting system [22, 24].

Hence, in this paper we consider the problem designing a nonlinear intervention strategy based on feedback linearization for biological phenomena modeled by S-systems. In this proposed algorithm, the control variables are designed such that an integral action is added to the system. The main advantage of the integral action is in improving the steady state performance of the closed-loop system. As a case study, the proposed intervention strategy is applied to a glycolytic-glycogenolytic pathway model. The glycolytic-glycogenolytic pathway model is selected as it plays an important role in cellular energy generation when the level of glucose in the blood is low (fasting state) and glycogen has to be broken down to provide the substrate to run glycolysis. By controlling the glycogenolytic reaction, one can exert control over whether glycolysis will run or not under low-glucose conditions.

This paper is organized as follows. In Section 2, the S-system model is presented and the control problem is formulated. In Section 3, some mathematical preliminaries as well as the feedback linearizable control scheme are presented. In Section 4, the glycolytic-glycogenolytic pathway model is considered as a case study. Finally, concluding remarks and possible future research directions are outlined in Section 5.

## 2. S-System Presentation and Problem Formulation

 Consider the following S-system model [25]:

(1) $$\begin{array}{c} {\dot{x}}_{i}=\alpha_{i}\prod_{j=1}^{N+m}x_{j}^{\theta_{ij}}-\beta_{i}\prod_{j=1}^{N+m}x_{j}^{\mu_{ij}},\;i=1,2,\ldots ,N, \end{array}$$

where *α*_*i*_ > 0 and *β*_*i*_ > 0 are rate coefficients and *θ*_*ij*_ and *μ*_*ij*_ are kinetic orders and there exist *N* + *m* variables (genes/metabolites) where the first *N* variables are dependent and the remaining *m* variables are independent variables. Assume that *p* out of the *N* dependent variables are target (or output) variables (i.e., genes/metabolites that need to be regulated to some desired final values), where these output variables are defined as

(2) $$\begin{array}{c} y_{j}=x_{i},\;j=1,\ldots ,p, \end{array}$$

and *i* ∈ *𝒴* ⊂ {1,…, *N*}, where *𝒴* is the set of indices corresponding to the dependent variables that are selected as output variables. The steady-state analysis of the S-system model [1, 18] shows that when the number of dependent variables with prespecified desired values is equal to the number of independent variables (which means that we have enough degrees of freedom), the above S-system model equations will have a unique steady-state solution under the nonsingularity assumption. Hence, in order to control the expressions/concentrations of the target variables, we consider an integral control approach where the following *r* equations are added to the above S-system:

(3) $$\begin{array}{c} {\dot{x}}_{i}=u_{j},\;j=1,\ldots ,p, \end{array}$$

where *i* ∈ *𝒰* ⊂ {*N* + 1,…, *N* + *m*}, where *𝒰* is the set of indices corresponding to the independent variables that are used as control variables. This means that *r* out of the *m* independent variables will be used as control variables, and the overall system will have *p* inputs and *p* outputs. It should be noted that the formulation above can be easily extended to deal with systems having more inputs than outputs. Let us denote by *𝒳* = {1,…, *N*} + *𝒰*, where *𝒳* corresponds to the indices of all variables except the independent variables that are not used as control variables. Here, it is assumed that the values (expressions/concentrations) of the independent variables that are not used as control variables are known constants (i.e., *x*_*i*_ = *δ*_*i*_, *i* ∈ {*N* + 1,…, *N* + *m*} − *𝒰*, where *δ*_*i*_ are known constants) [6].

**Figure 1:**
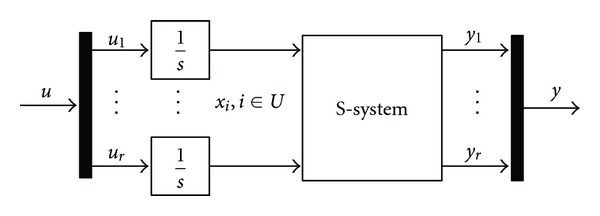
S-system with integral control architecture.

Figure 1 shows the S-system (1) augmented by the integral control. The S-system with integral control (1)–(3) can be written in the form

(4) $$\begin{array}{c} \dot{x}=f\left(x\right)+g\left(x\right)u,\\ y=h\left(x\right), \end{array}$$

where *x* = [*x*_*i*_]^*T*^ ∈ ℝ^*N*+*p*^, *i* ∈ *𝒳*, *u* = [*u*_1_,…, *u*_*p*_]^*T*^ ∈ ℝ^*p*^, *y* = [*y*_1_,…, *y*_*p*_]^*T*^ ∈ ℝ^*p*^ and

(5) $$\begin{array}{c} f\left(x\right)=\left[\begin{array}{c} \alpha_{1}\prod_{j=1}^{N+m}x_{j}^{\theta_{1j}}-\beta_{1}\prod_{j=1}^{N+m}x_{j}^{\mu_{1j}}\\ \vdots \\ \alpha_{N}\prod_{j=1}^{N+m}x_{j}^{\theta_{Nj}}-\beta_{N}\prod_{j=1}^{N+m}x_{j}^{\mu_{Nj}}\\ 0\\ \vdots \\ 0 \end{array}\right]\begin{array}{c} \}p, \end{array}\\ g\left(x\right)=\left[\begin{array}{c} 0_{N\times p}\\ I_{p\times p} \end{array}\right],\;h\left(x\right)={\left[x_{i}\right]}^{T},\;i\in 𝒴, \end{array}$$

which can be expressed as

(6) $$\begin{array}{c} \dot{x}=f\left(x\right)+\sum_{i=1}^{p}g_{i}\left(x\right)u_{i}, \end{array}$$

(7) $$\begin{array}{c} y_{i}=h_{i}\left(x\right), \end{array}$$

where *g*_*i*_(*x*) = [0_1_,0_2_,…,0_*N*+*i*−1_,1_*N*+*i*_,0_*N*+*i*+1_,…,0_*N*+*p*_]^*T*^, for *i* = 1,…, *p*.

**Problem Formulation**

Suppose that the outputs of the S-system (1) are initially at the steady-state condition *y*_0_*j*__, *j* = 1,…, *p*. Let us denote by *y*_*d*_*j*__, *j* = 1,…, *p*, the desired final steady state values of the output (target) variables. Then, the main goal of the feedback linearizable controller is to determinate the control inputs *u*_*j*_, *j* = 1,…, *p*, that can guide the target variables from the initial steady-state condition to the final one [18].

## 3. Feedback Linearizable Intervention

 Here, we show how feedback linearization can be utilized to design a nonlinear intervention strategy to control biological phenomena modeled by S-systems. Feedback linearization can be used to obtain a linear relationship between the output vector *y* and a new input vector *v*, by making a right choice of the linearizing law. Once the equivalent model becomes linear, we may design a dynamic control law-based classical linear control theory. Before starting the development of this control technique, it is important to introduce the following mathematical preliminaries [20–22].

### 3.1. Mathematical Preliminaries

 Let the vector function *f* : *ℜ* ^*n*^ → *ℜ* ^*n*^ be a vector field in *ℜ* ^*n*^. The vector function *f*(*x*) is called a smooth vector function if it has continuous partial derivatives of any required order [26]. Given a scalar function *h*(*x*) and a vector field *f*(*x*), we define a new scalar function *L*_*f*_ *h*, called the Lie derivative of *h* with respect to *f*, as follows.

**Definition 1 (see [26])**

Let *h* : *ℜ* ^*n*^ → *ℜ* be a smooth scalar function, and *f* : *ℜ* ^*n*^ → *ℜ* ^*n*^ be a smooth vector field on *ℜ* ^*n*^, then the Lie derivative of *h* with respect to *f* is a scalar function defined by *L*_*f*_ *h* = ∇*hf*.

Thus, the Lie derivative *L*_*f*_ *h* is simply the directional derivative of *h* along the direction of the vector *f*. Repeated Lie derivatives can be defined recursively as follows:

(8) $$\begin{array}{c} L_{f}^{\left(0\right)}h=h,\\ L_{f}^{\left(i\right)}h=L_{f}\left(L_{f}^{\left(i-1\right)}h\right)=\nabla \left(L_{f}^{\left(i-1\right)}h\right)f,\;\text{for}\;\;i=1,2,\ldots . \end{array}$$

Similarly, if *g* is another vector field, then the scalar function *L*_*g*_ *L*_*f*_ *h*(*x*) can be described as

(9) $$\begin{array}{c} L_{g}L_{f}h=\nabla \left(L_{f}h\right)g. \end{array}$$

**Definition 2 (see [26])**

Let *f* and *g* be two vector fields on *ℜ* ^*n*^. The Lie bracket of *f* and *g* is a third vector field defined by

(10) $$\begin{array}{c} \left[f,g\right]=\nabla gf-\nabla fg, \end{array}$$

where the Lie bracket [*f*, *g*] is commonly written as ad_*f*_ *g* (where ad stands for “adjoint”).

Repeated Lie brackets can then be defined recursively by ad_*f*_^(0)^ *g* = *g*,…, ad_*f*_^(*i*)^ *g* = [*f*, ad_*f*_^(*i*−1)^ *g*].

### 3.2. Feedback Linearizable Controller

 Consider the S-system model (6). Differentiating the *j*th output *y*_*j*_ of this system with respect to time, we get

(11) $$\begin{array}{c} {\dot{y}}_{j}=L_{f}h_{j}\left(x\right)+\sum_{i=1}^{p}\left(L_{g_{i}}h_{j}\left(x\right)\right)u_{i}, \end{array}$$

for *j* = 1,2, 3,…*p*. Note in (7) that if each of the *L*_*g*_*i*__ *h*_*j*_(*x*) = 0, then the inputs do not appear in the equation. Define *γ*_*j*_ to be the smallest integer such that at least one of the inputs appears in *y*_*j*_^(*γ*_*j*_)^, that is

(12) $$\begin{array}{c} y_{i}^{\left(\gamma_{j}\right)}=L_{f}^{\left(\gamma_{j}\right)}h_{j}\left(x\right)+\sum_{i=1}^{p}L_{g_{i}}\left(L_{f}^{\left(\gamma_{j}-1\right)}h_{j}\left(x\right)\right)u_{i}, \end{array}$$

with at least one of the *L*_*g*_*i*__(*L*_*f*_^(*γ*_*j*_−1)^ *h*_*j*_) ≠ 0, for some *x*. Let the *p* × *p* matrix *D*(*x*) be defined as

(13) $$\begin{array}{c} D\left(x\right)=\left(\begin{array}{ccc} L_{g_{1}}L_{f}^{\left(\gamma_{1}-1\right)}h_{1} & L_{g_{2}}L_{f}^{\left(\gamma_{1}-1\right)}h_{1} & \cdots \;L_{g_{p}}L_{f}^{\left(\gamma_{1}-1\right)}h_{1}\\ L_{g_{1}}L_{f}^{\left(\gamma_{2}-1\right)}h_{2} & L_{g_{2}}L_{f}^{\left(\gamma_{2}-1\right)}h_{2} & \cdots \;L_{g_{p}}L_{f}^{\left(\gamma_{2}-1\right)}h_{2}\\ \vdots & \vdots & \vdots \\ L_{g_{1}}L_{f}^{\left(\gamma_{p}-1\right)}h_{p} & L_{g_{2}}L_{f}^{\left(\gamma_{p}-1\right)}h_{p} & \cdots \;L_{g_{p}}L_{f}^{\left(\gamma_{p}-1\right)}h_{p} \end{array}\right). \end{array}$$

Based on the above definitions, the relative degree for multiinput multioutput (MIMO) systems is defined next.

**Definition 3 (see [27])**

The system (6)-(7) is said to have vector relative degree *γ*_1_, *γ*_2_,…, *γ*_*p*_ at *x*_0_ if *L*_*g*_*i*__ *L*_*f*_^(*k*)^ *h*_*i*_(*x*) ≡ 0, 0 ≤ *k* ≤ *γ*_*i*_ − 2, for *i* = 1,…, *p* and the matrix *D*(*x*_0_) is nonsingular.

If a system has well-defined vector relative degree, then (12) can be expressed as

(14) $$\begin{array}{c} {\left[y_{1}^{\left(\gamma_{1}\right)},y_{2}^{\left(\gamma_{2}\right)},\ldots ,y_{p}^{\left(\gamma_{p}\right)}\right]}^{T}=\xi \left(x\right)+D\left(x\right)u, \end{array}$$

where

(15) $$\begin{array}{c} \xi \left(x\right)={\left[L_{f}^{\left(\gamma_{1}\right)}h_{1}\left(x\right),L_{f}^{\left(\gamma_{2}\right)}h_{2}\left(x\right),\ldots ,L_{f}^{\left(\gamma_{p}\right)}h_{p}\left(x\right)\right]}^{T}. \end{array}$$

Since *D*(*x*_0_) is nonsingular, it follows that *D*(*x*) ∈ *ℜ* ^*p*×*p*^ is bounded away from nonsingularity for *x* ∈ *U*, a neighborhood *U* of *x*_0_. Then, the state feedback control law

(16) $$\begin{array}{c} u=D{\left(x\right)}^{-1}\left(-\xi \left(x\right)+v\right) \end{array}$$

yields the linear closed-loop system

(17) $$\begin{array}{c} y_{i}^{\left(\gamma_{j}\right)}=v_{i}. \end{array}$$

**Figure 2:**
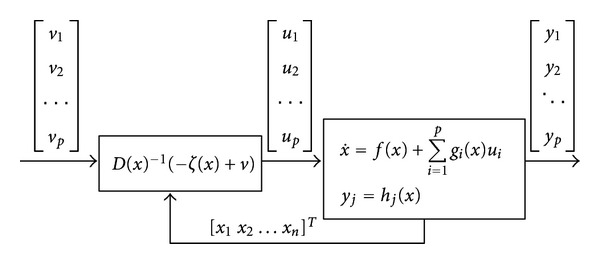
Diagram block of the linearizable system.

The block diagram of the linearized system is shown in Figure 2. 

Feedback linearization transforms the system into a linear system where linear control approaches can be applied. Here, *v* represents the new input vector of the linearized system.

In the case the system has vector relative degree, where *γ*_1_ + ⋯+*γ*_*p*_ = *n*, the nonlinear system can be converted into a controllable linear system, where the feedback control law is defined in (16) and the coordinate transformation is *ξ*(*x*) = [*L*_*f*_^(*j*)^ *h*_*i*_(*x*)]^*T*^, 0 ≤ *j* ≤ *γ*_*i*_ − 1, 0 ≤ *i* ≤ *p*. Let the following distributions be defined as [27]

(18) G0(x)=span⁡{g1(x),…,gp(x)},G1(x)=span⁡{g1(x),…,gp(x),adfg1,…,adfgp(x)},⋮Gi(x)=span⁡{adf(k)gi(x):0≤k≤i, 0≤j≤p},

for *i* = 1,…, *n* − 1, then we have the following result.

**Proposition 4 (see [27])**

Suppose that the matrix *g*(*x*_0_) has rank *p*. Then, there exist *p* functions *λ*_1_,…, *λ*_*p*_, such that the system

(19) $$\begin{array}{c} \dot{x}=f\left(x\right)+g\left(x\right)u,\\ y=\lambda \left(x\right), \end{array}$$

has vector relative degree (*γ*_1_,…, *γ*_*p*_) with *γ*_1_ + *γ*_2_ + ⋯+*γ*_*p*_ = *n* if

- *for each 0 ≤ *i* ≤ *n* − 1 the distribution *G*_*i*_ has constant dimension in the neighborhood *U* of *x*_0_; *
- *the dimension *G*_*n*−1_ has dimension *n*; *
- *for each 0 ≤ *i* ≤ *n* − 2 the dimension *G*_*i*_ is involutive. *

The proof of this proposition can be found in [27].

The new control vector *v* = [*v*_1_,…,*v*_*p*_]^*T*^ is designed based on the desired closed-loop response, which can be written as

(20) $$\begin{array}{c} v_{j}=y_{d_{j}}^{\left(\gamma_{j}\right)}+k_{\gamma_{j}-1}\left(y_{d_{j}}^{\left(\gamma_{j}-1\right)}-y_{j}^{\left(\gamma_{j}-1\right)}\right)+\cdots +k_{1}\left(y_{d_{j}}-y_{j}\right) \end{array}$$

for *j* = 1,…, *p*, where {*y*_*d*_*j*__, *y*_*d*_*j*__^(1)^,…, *y*_*d*_*j*__^(*γ*_*j*_−1)^, *y*_*d*_*j*__^(*γ*_*j*_)^} denotes the desired reference trajectories for the outputs. The proportional gains are chosen such that the following polynomial is a Hurwitz polynomial [28]:

(21) $$\begin{array}{c} s^{\gamma_{j}}+k_{\gamma_{j}-1}s^{\gamma_{j}-1}+\cdots +k_{2}s+k_{1}=0. \end{array}$$

**Figure 3:**
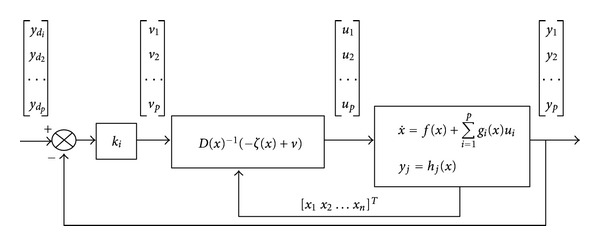
Closed loop of the linearizable system.

The block diagram of the closed-loop system in the feedback linearizable form is shown in Figure 3.

## 4. Case Study

**Figure 4:**
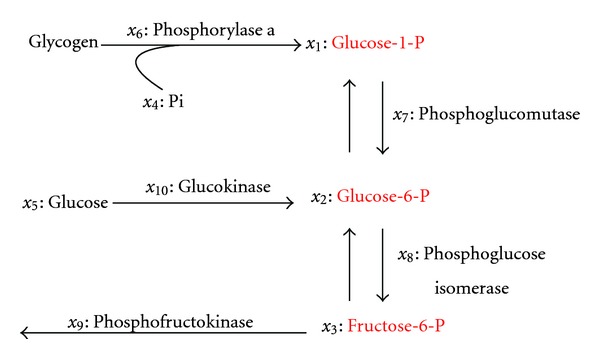
Glycolytic-glycogenolytic pathway [29].

In this section, we demonstrate the efficacy of the feedback linearizable intervention approach described in this paper by applying it to a well-studied biological pathway model representing the glycolytic-glycogenolytic pathway shown in Figure 4 [17, 29]. Glycolysis is the process of breaking up a six-carbon glucose molecule into two molecules of a three-carbon compound, and glycogenolysis is the process by which the stored glycogen in the body is broken up to meet the needs for glucose. In glycogenolysis, the phosphorylase enzyme acts on the polysaccharide glycogen to reduce its length by one glucose unit. The glucose unit is released as a glucose-1 phosphate. The glycolytic-glycogenolytic pathway can be mathematically represented by the following S-system model:

(22) $$\begin{array}{c} {\dot{x}}_{1}=\alpha_{1}x_{4}^{\theta_{14}}x_{6}^{\theta_{16}}-\beta_{1}x_{1}^{\mu_{11}}x_{2}^{\mu_{12}}x_{7}^{\mu_{17}},\\ {\dot{x}}_{2}=\alpha_{2}x_{1}^{\theta_{21}}x_{2}^{\theta_{22}}x_{5}^{\theta_{25}}x_{7}^{\theta_{27}}x_{10}^{\theta_{210}}-\beta_{2}x_{2}^{\mu_{22}}x_{3}^{\mu_{23}}x_{8}^{\mu_{28}},\\ {\dot{x}}_{3}=\alpha_{3}x_{2}^{\theta_{32}}x_{3}^{\theta_{33}}x_{8}^{\theta_{38}}-\beta_{3}x_{3}^{\mu_{33}}x_{9}^{\mu_{39}}. \end{array}$$

In this case, *N* = 3, *m* = 7 and the parameter are defined as *α*_1_ = 0.077884314, *θ*_14_ = 0.66, *θ*_16_ = 1, *β*_1_ = 1.06270825, *μ*_11_ = 1.53, *μ*_12_ = −0.59, *μ*_17_ = 1, *α*_2_ = 0.585012402, *θ*_21_ = 0.95, *θ*_22_ = −0.41, *θ*_25_ = 0.32, *θ*_27_ = 0.62, *θ*_210_ = 0.38, *β*_2_ = *α*_3_ = 0.0007934561, *μ*_22_ = *θ*_32_ = 3.97, *μ*_23_ = *θ*_33_ = −3.06, *μ*_28_ = *θ*_38_ = 1, *β*_3_ = 1.05880847, *μ*_33_ = 0.3, and *μ*_39_ = 1. Here, the model variables are defined as follows: *x*_1_ is glucose-1-P, *x*_2_ is glucose-6-P, *x*_3_ is fructose-6-P, *x*_4_ is inorganic phosphate ion, *x*_5_ is glucose, *x*_6_ is phosphorylase *a*, *x*_7_ is phosphoglucomutase, *x*_8_ is phosphoglucose isomerase, *x*_9_ is phosphofructokinase, and *x*_10_ is glucokinase.

For this model, the metabolites *x*_4_ through *x*_10_ are defined as independent variables, which are the variables that are not affected by other variables, and the metabolites *x*_1_ through *x*_3_ are defined as the dependent variables, which are the primary variables of interest that we wish to control. Here, we choose the independent variables *x*_4_, *x*_5_, and *x*_8_ as manipulated or control variables, as shown in Figure 4, as they can affect the production of the dependent variables *x*_1_, *x*_2_, and *x*_3_. Also, we choose to keep the independent variables *x*_6_, *x*_7_, *x*_9_, and *x*_10_ fixed ignoring their effect on the controlled variables, and assuming that the controller only uses the independent variables *x*_4_, *x*_5_, and *x*_8_ to control the dependent variables *x*_1_, *x*_2_, and *x*_3_. The independent variables have the following values *x*_4_ = 10, *x*_5_ = 5, *x*_6_ = 3, *x*_7_ = 40, *x*_8_ = 136, *x*_9_ = 2.86, and *x*_10_ = 4. Here, we try to control *x*_1_, *x*_2_, and *x*_3_ by manipulating *x*_4_, *x*_5_, and *x*_8_, so we have

(23) $$\begin{array}{c} y_{1}=x_{1},\\ y_{2}=x_{2},\\ y_{3}=x_{3},\\ {\dot{x}}_{4}=u_{1},\\ {\dot{x}}_{5}=u_{2},\\ {\dot{x}}_{8}=u_{3}, \end{array}$$

and all other *x*_*i*_′*s* for *i* = 6,7, 9, and 10 are kept fixed. The initial values of the outputs *y*_1_, *y*_2_, and *y*_3_ are selected as 0.067, 0.465, and 0.150, respectively, and the desired reference outputs are selected as *y*_*d*1_ = 0.2, *y*_*d*2_ = 0.5, and *y*_*d*3_ = 0.4.

Hence, the overall system can be expressed in the form of (6), where

(24) f(x)=[a1x4θ14−b1x1μ11x2μ12a2x1θ21x2θ22x5θ25−b2x2μ22x3μ23x8μ28a3x2θ32x3θ33x8θ38−b3x3μ33000],g(x)=[g1(x),g2(x),g3(x)]=[000100000010000001]T,h(x)=[h1(x)h2(x)h3(x)]=[100000010000001000],

where *a*_1_ = *α*_1_ *x*_6_^*θ*_16_^, *a*_2_ = *α*_2_ *x*_7_^*θ*_27_^ *x*_10_^*θ*_210_^, *a*_3_ = *α*_3_, *b*_1_ = *β*_1_ *x*_7_^*μ*_17_^, *b*_2_ = *β*_2_, and *b*_3_ = *β*_3_ *x*_9_^*μ*_39_^.

Based on the S-system model describing the glycolytic-glycogenolytic pathway, it can be verified that the outputs need to be differentiated twice with respect to time so that the input variables (*u*_1_, *u*_2_, or *u*_3_) appear in the expressions of differentiated outputs, as follows:

(25) y1(2)=Lf(2)h1(x)+Lg1(Lfh1(x))u1,y2(2)=Lf(2)h2(x)+Lg2(Lfh2(x))u2+Lg3(Lfh2(x))u3,y3(2)=Lf(2)h3(x)+Lg3(Lfh3(x))u3,

where

(26) Lf(2)h1(x)=b12μ11x12μ11−1x22μ12−b1a1μ11x1μ11−1x2μ12x4θ14+b1b2μ12x2μ12+μ22−1x1μ11x3μ23x8μ28−b1a2μ12x2μ12+θ21−1x1μ11+θ21x5θ25,Lf(2)μ2(x)=a1a2θ21x1θ21−1x2θ22x4θ14x5θ25−a2b1x1θ21+μ11−1x2θ22+μ12x5θ25+a22θ22x22θ22−1x12θ21x5θ25−a2b2x2θ22+μ22−1x1θ21x3μ23x5θ25x8μ28−b2a2μ22x1θ12x2μ22+θ22−1x3μ23x5θ25x8μ28+b22μ22x22μ22−1x32μ23x82μ28−b2a3μ23x2μ22+θ32x3μ23+θ33−1x8μ28+θ38+b2b3μ23x2μ22x3μ23+μ33−1x8μ28,Lf(2)μ3(x)=a3a2θ32x1θ21x2θ32+θ22−1x3θ33x5θ25x8θ38−a3b2θ32x2θ32+μ22−1x3θ33+μ23x8θ38+μ28+a32θ33x22θ32x32θ33−1x82θ38−a3b3θ33x2θ32x3θ33+μ33−1x8θ38−b3a3μ33x2θ32x3μ33+θ33−1x8θ38+b32μ33x32μ33−1,Lg1(Lfh1(x))=a1x4θ14−1,Lg2(Lfh2(x))=a2θ25x1θ21x2θ22x5θ25−1,Lg3(Lfh2(x))=−b2μ28x2μ22x3μ23x8μ28−1,Lg3(Lfh3(x))=a3θ38x2θ32x3θ33x8θ38−1.

Hence, in this case the system has vector relative degree *γ* = [*γ*_1_,*γ*_2_,*γ*_3_]^*T*^ = [2,2,2]^*T*^, and hence we have *γ*_1_ + *γ*_2_ + *γ*_3_ = 6.

The matrix form of the system of differential equations presented in (25) can be written in the form of (14), where

(27) $$\begin{array}{c} \xi \left(x\right)={\left[L_{f}^{\left(2\right)}h_{1}\left(x\right),L_{f}^{\left(2\right)}h_{2}\left(x\right),L_{f}^{\left(2\right)}h_{3}\left(x\right)\right]}^{T},\\ D\left(x\right)=\left(\begin{array}{ccc} L_{g_{1}}\left(L_{f}h_{1}\left(x\right)\right) & 0 & 0\\ 0 & L_{g_{2}}\left(L_{f}h_{2}\left(x\right)\right) & L_{g_{3}}\left(L_{f}h_{2}\left(x\right)\right)\\ 0 & 0 & L_{g_{3}}\left(L_{f}h_{3}\left(x\right)\right) \end{array}\right). \end{array}$$

The matrix *D*(*x*) is invertible if the following condition is satisfied:

(28) $$\begin{array}{c} L_{g_{1}}\left(L_{f}h_{1}\left(x\right)\right)\times L_{g_{2}}\left(L_{f}h_{2}\left(x\right)\right)\times L_{g_{3}}\left(L_{f}h_{3}\left(x\right)\right)\neq 0. \end{array}$$

Based on (25), it can be seen that the control variables *u*_1_ and *u*_2_ appear only in the expressions of *y*_1_^(2)^ and *y*_2_^(2)^, respectively. However, *u*_3_ appears in the expressions of *y*_2_^(2)^ and *y*_3_^(2)^. Hence, *u*_1_ and *u*_3_ need to be used to control *y*_1_ and *y*_3_, respectively, and both *u*_2_ and *u*_3_ are needed to control *y*_2_.

Hence, the control laws based on (16) can be expressed as

(29) $$\begin{array}{c} u_{1}=\frac{\left(-L_{f}^{\left(2\right)}h_{1}\left(x\right)+v_{1}\right)}{L_{g_{1}}\left(L_{f}h_{1}\left(x\right)\right)},\\ u_{2}=\frac{\left(-L_{f}^{\left(2\right)}h_{2}\left(x\right)-L_{g_{3}}\left(L_{f}h_{3}\left(x\right)\right)u_{3}+v_{2}\right)}{L_{g_{2}}\left(L_{f}h_{2}\left(x\right)\right)},\\ u_{3}=\frac{\left(-L_{f}^{\left(2\right)}h_{3}\left(x\right)+v_{3}\right)}{L_{g_{3}}\left(L_{f}h_{3}\left(x\right)\right)}. \end{array}$$

Substituting the expressions of the control variables (29) in (25), we obtain the following decoupled linear system:

(30) $$\begin{array}{c} y_{1}^{\left(2\right)}=v_{1},\\ y_{2}^{\left(2\right)}=v_{2},\\ y_{3}^{\left(2\right)}=v_{3}. \end{array}$$

The new control variables *v*_*j*_, for *j* = 1,2, 3, need to be designed so that the target variables *y*_*j*_ track some desired reference trajectories, *y*_*d*_*j*__.

Using (20), the new control variables *v*_*j*_, for *j* = 1,2, 3, are found to be

(31) $$\begin{array}{c} v_{1}={\ddot{y}}_{d_{1}}+k_{1}\left({\dot{y}}_{d_{1}}-{\dot{y}}_{1}\right)+k_{11}\left(y_{d_{1}}-y_{1}\right),\\ v_{2}={\ddot{y}}_{d_{2}}+k_{2}\left({\dot{y}}_{d_{2}}-{\dot{y}}_{2}\right)+k_{21}\left(y_{d_{2}}-y_{2}\right),\\ v_{3}={\ddot{y}}_{d_{3}}+k_{3}\left({\dot{y}}_{d_{3}}-{\dot{y}}_{3}\right)+k_{31}\left(y_{d_{3}}-y_{3}\right). \end{array}$$

The new control components, *v*_1_, *v*_2_, and *v*_3_, are defined in (31), where the parameters are selected as *k*_1_ = 1, *k*_11_ = 5, *k*_2_ = 10^−3^, *k*_21_ = 20, *k*_3_ = 3, and *k*_31_ = 5.

**Figure 5:**
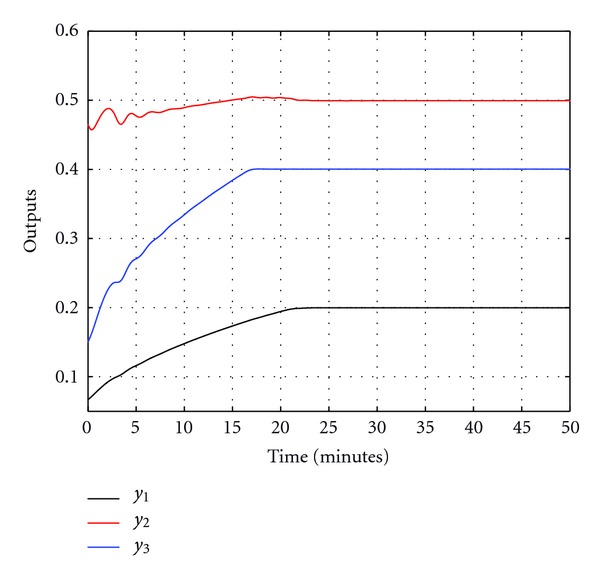
Closed-loop outputs for constant reference signals.

**Figure 6:**
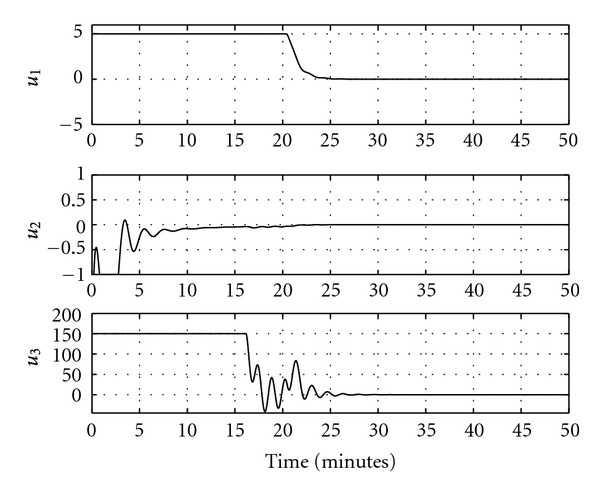
Control signals for constant reference signals.

**Figure 7:**
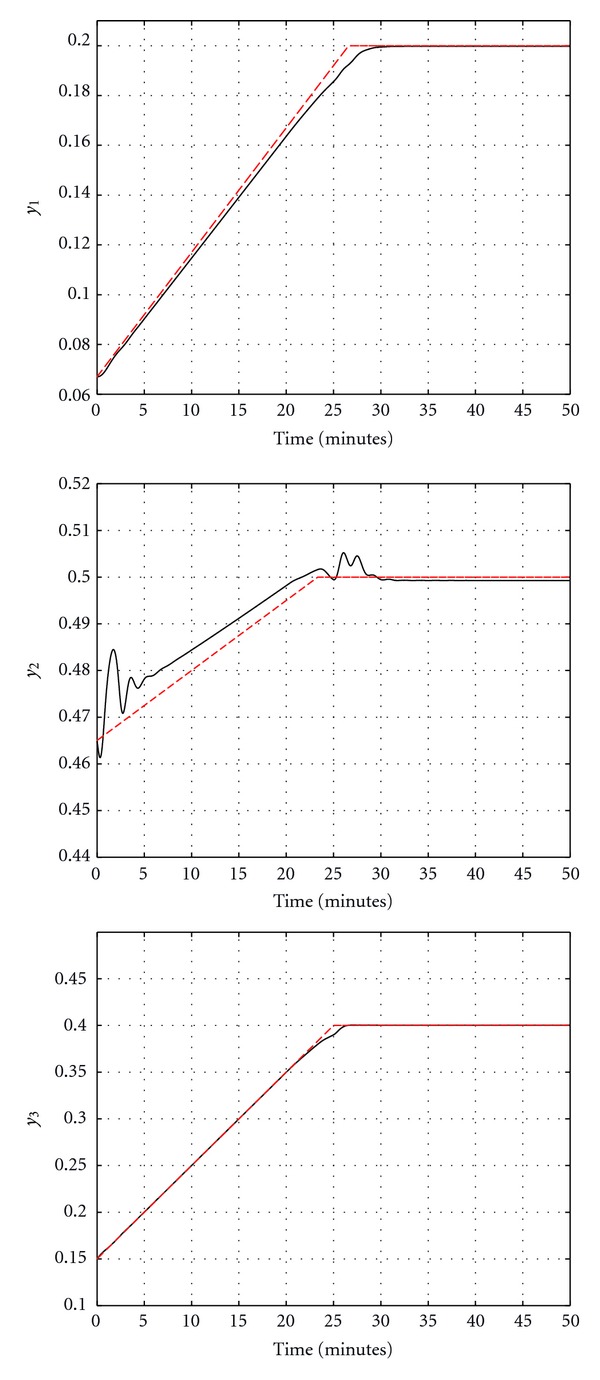
Output response for closed-loop tracking.

**Figure 8:**
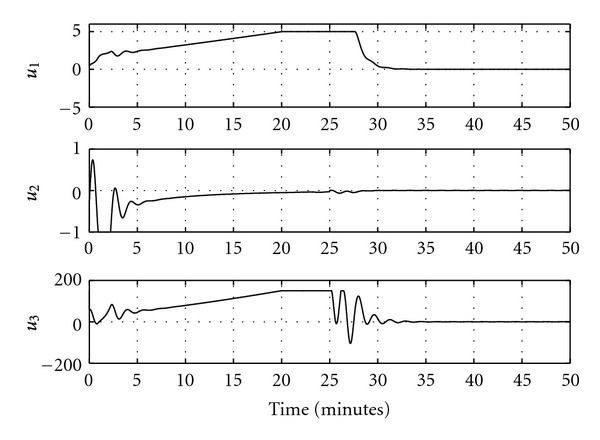
Control signals for closed-loop tracking.

Figures 5 and 6 show the output response and the control input signals when the feedback linearizable controller is applied. It is clear from Figure 5 that the system outputs converge to their desired values. Another simulation study is implemented for a different reference trajectory, where the value of the reference signal increases linearly before saturating at the desired final value. The closed-loop output response in this case is shown in Figure 7 and the control signals are shown in Figure 8. It is clear from Figure 7 that the feedback linearizable controller is driving the target variables to track the desired reference trajectories.

To study the robustness properties of the feedback linearizable controller, similar simulation studies have been conducted when the parameters *μ*_22_ and *β*_2_ are varied within 10% of their nominal values. It has been found that the closed-loop system is stable only for parameter variations within 1% and with unacceptable performance. This agrees with our earlier assumption that full system knowledge is needed for proper operation of the feedback linearizable controller.

## 5. Conclusion

 In this paper, feedback linearizable control has been applied for intervention of biological phenomena modeled in the S-system framework. As a case study, the glycogenolytic-glycolytic pathway model has been used to demonstrate the efficacy of feedback linearization in controlling biological phenomena modeled by S-system. One main drawback of this approach is that it assumes full knowledge of the biological system model. Usually, the S-system model does not perfectly represent the actual dynamics of the biological phenomena. Hence, one future research direction is to develop an adaptive intervention strategy that is capable of controlling the biological system even in the presence of model uncertainties. Another future research direction is to develop intervention techniques that take into account additional constraints due to the nature of the drug injection process. Definitely, incorporating such knowledge from medical practitioners would require imposing constraints on the magnitude, duration, and possibly the rate of change of the injected drug into the design of intervention technique.
